# Culture optimization of*Escherichia coli *for expression ofgE proteinfrombovine herpesvirus 1 and 5

**DOI:** 10.1186/1753-6561-8-S4-P167

**Published:** 2014-10-01

**Authors:** Maraninchi Roberta Silveira, Cláudia Pinho Hartleben, Leonardo Garcia Monte, Gizele Lima de Sá, Fabrício Conceição, Fabrício Souza Campos, Paulo Roehe, Bianca Sica Siedler

**Affiliations:** 1 Universidade Federal de Pelotas, Centro de Desenvolvimento Tecnológico, Biotecnologia, Laboratório de Imunodiagnóstico, Capão do Leão, RS, Brazil; 2 Universidade Federal de Pelotas, Capão do Leão, Brazil; 3 Universidade Federal do Rio Grande do Sul, Instituto de Biociências, Laboratório de Virologia, Capão do Leão, RS, Brazil; 4 Universidade Federal de Pelotas, Centro de Desenvolvimento Tecnológico, Biotecnologia, Laboratório de Imunodiagnóstico, Capão do Leão, RS, Brazil

## Background

The use of *Escherichia coli *for the production of recombinant proteins is an established strategy to obtain biotechnological tools. However, the recombinant protein expression is dependent on temperature, bacterial culture time, induction period, nutrients, plasmid characteristics, and insert itself. Thus, the aim of this study was optimize the expression of recombinant gE protein (rgE) from Bovine Herpesvirus types 1 and 5.

## Methods

The expression at various post-induction time-points and during growth at two temperatures was performed in order to standardize the conditions that *E. coli *maximizes rgE expression. A recombinant vector pAE/gE containing a consensus sequence between BoHV-1 and BoHV-5 was used to heat-shock transform *E. coli *strain BL21 Star™ (DE3). The transformation product was seeded on solid Luria Bertani (LB) medium containing ampicillin (100 µg/mL). After, the colonies selected were grown in LB medium (1 mL) and incubated at 37 °C for 16 h. Then, 0.5 mL of this culture was transferred to 10 mL of LB medium and incubated at 37 °C again to reach the exponential phase of bacterial growth (OD_600 _0.6 - 0.8). The bacterial culture was induced with 0.6 mM IPTG for periods of 4, 6 and 12 h at 25 ºC or 37 ºC. Aliquots from each culture condition tested were collected and analyzed by SDS-PAGE and Western Blot (WB).

## Results and conclusions

The finding of this study indicated that rgE protein was successfully expressed after induction for 12 h at 25 °C. These conditions will be used to obtain rgE lots for development of immunodiagnostic assays of bovine Herpesvirus type 1 and 5.
